# Current Trends in Balance Rehabilitation for Stroke Survivors: A Scoping Review of Experimental Studies

**DOI:** 10.3390/ijerph20196829

**Published:** 2023-09-26

**Authors:** Júlia Saraiva, Gonçalo Rosa, Sónia Fernandes, Júlio Belo Fernandes

**Affiliations:** 1 Department of Nursing, Hospital Garcia de Orta, 2805-267 Almada, Portugal; grosa@egasmoniz.edu.pt; 2 Nurs* Lab, Caparica, 2829-511 Almada, Portugal; sfernandes@egasmoniz.edu.pt (S.F.); jfernandes@egasmoniz.edu.pt (J.B.F.); 3 Egas Moniz Center for Interdisciplinary Research (CiiEM), Egas Moniz School of Health & Science, Caparica, 2829-511 Almada, Portugal

**Keywords:** stroke, stroke rehabilitation, rehabilitation, postural balance, exercise therapy, exercise

## Abstract

Balance impairment is a common consequence of a stroke, which can significantly hinder individuals’ participation in daily activities, social interactions, and leisure pursuits and their ability to return to work. Rehabilitation is vital for minimizing post-stroke sequelae and facilitating the recovery of patients. This review aims to identify current trends in balance rehabilitation of stroke survivors. This Scoping review followed Arksey and O’Malley’s methodological framework. The literature search was conducted in electronic databases, including CINAHL Complete, MEDLINE Complete, and Nursing & Allied Health Collection. The search was performed in March 2023, and the inclusion criteria were articles published in English or Portuguese between 2013 and 2023. A total of 446 articles were identified. After selecting and analyzing the reports, fourteen publications were included in this review. Seven distinct categories of balance rehabilitation interventions were identified, covering various approaches. These categories included conventional rehabilitation exercises, gym-based interventions, vibration therapy, rhythmic auditory stimulation training, boxing therapy, dual-task training, and technology-based rehabilitation interventions. Each of these methods presents unique benefits and can significantly impact the recovery of balance in stroke survivors, enhancing their overall well-being and functional capacity.

## 1. Introduction

Stroke is the second leading cause of death worldwide and significantly contributes to long-term disability [1,2]. With an aging population and improved survival rates, the number of individuals affected by stroke is rising [3]. Recent global data from 2022 indicate a 50% increase in stroke risk over the past 17 years, with an estimated 1 in 4 people experiencing a stroke in their lifetime. Additionally, between 1990 and 2019, there was a 70% increase in stroke incidence and a 43% increase in stroke-related deaths [4].

Stroke is characterized by the rapid onset of focal or global cerebral function disorders, lasting more than 24 hours or leading to death, with no apparent cause other than vascular origin [5].

Functional changes are common among stroke survivors [6]. Cognitive and motor impairments affecting balance, coordination, proprioception, muscle tone, muscle strength, and gait are prevalent sequelae of stroke [6,7].

Stroke deficits are devastating, causing not only many physical and cognitive impairments, but also emotional. A stroke can affect the individual’s health and overall well-being and ability to lead an independent and fulfilling life [8,9].

Previous studies have reported balance impairment in up to 83% of stroke survivors [10]. These impairments hinder participation in daily activities, social interactions, and leisure pursuits and returning to work post stroke [11,12].

Furthermore, balance impairments contribute significantly to negative consequences following a stroke, including an increased risk of falls [13,14]; fall-related injuries, such as fractures [15]; fear of falling [16]; and even mortality [17]. Additionally, a previous study found that balance impairment during the acute phase of stroke predicted cognitive impairment one year post stroke [18].

These findings underscore the importance of addressing balance impairments in stroke rehabilitation to mitigate adverse outcomes. Research has shown that the recovery rate tends to be most rapid within the first few weeks after a stroke. During this critical period, targeted balance interventions can impact the individual’s overall recovery trajectory. However, it is critical to recognize that the window of opportunity is time sensitive, as studies have indicated a considerable decline in rehabilitation gains between 1 and 3 months post stroke [19,20,21]. Furthermore, there is a significant drop in potential benefits after six months [21].

Incorporating balance-focused rehabilitation early in the stroke recovery journey can lead to more favorable outcomes, enhance functional independence, and ultimately improve the quality of life for stroke survivors [20,21]. These insights underscore the urgency of adopting evidence-based rehabilitation strategies that prioritize balance enhancement in stroke care protocols.

Rehabilitation plays a vital role in helping stroke survivors regain functional capacity. Engaging in rehabilitation programs that prioritize balance and muscle-strengthening training is a crucial factor that directly influences an individual’s recovery [22,23,24]. Implementing a rehabilitation program that includes balance-promoting exercises is essential, as good balance is a prerequisite for regaining the ability to walk independently and perform activities of daily living [25].

Previous research has indicated that health professionals commonly prescribe conventional rehabilitation exercises to enhance balance in stroke survivors [26,27]. Furthermore, akin to various other functional rehabilitation programs for stroke, these exercises for balance improvement demand rigorous and repetitive task-specific practice [28,29].

Several studies have consistently demonstrated the effectiveness of various interventions in enhancing balance among stroke survivors. These interventions range from traditional gym-based approaches [30,31,32] to innovative technologies, such as game-based virtual reality [33] or robot-assisted trunk control training [34]. This research highlights the importance of starting balance rehabilitation early, as soon as the individual’s medical condition stabilizes.

Healthcare professionals play a pivotal role in facilitating the recovery and rehabilitation of stroke survivors. Central to this responsibility is selecting the most appropriate intervention to optimize everyone’s rehabilitation journey. This selection process demands a holistic approach that draws upon a deep understanding of the available evidence concerning the effectiveness of diverse interventions. Moreover, it necessitates integrating patient preferences, assessing available resources, and an astute awareness of the unique clinical context in which rehabilitation is administered [35,36].

Evidence synthesis has become invaluable for healthcare professionals in this complex landscape. Such synthesis, often accomplished through methods like scoping reviews, provides a structured and comprehensive approach to navigate the literature on a specific subject. Scoping reviews are especially beneficial for presenting an overview of a vast and diverse body of research, which is particularly useful for broad topics, such as stroke survivor rehabilitation [37,38].

Scoping reviews help distill complex information into manageable insights, shedding light on emerging trends, gaps in knowledge, and potential areas for further investigation. Therefore, this review aims to identify current trends in balance rehabilitation of stroke survivors.

## 2. Methods

Given the study aim, we have chosen to conduct a scoping review. Researchers often choose scoping reviews when the review’s purpose is to identify knowledge gaps, delineate a body of literature, clarify concepts, or investigate research practices [37]. While scoping reviews are valuable, they can also serve as precursors to systematic reviews, helping to confirm the relevance of eligibility criteria and potential research questions [38]. Therefore, to identify current trends in balance rehabilitation for stroke survivors, we chose a scoping review as a valid and appropriate research method.

The present scoping review follows the methodological approach proposed by Arksey and O’Malley [37], which consists of five stages: (1) identifying research questions, (2) identifying relevant studies, (3) study selection, (4) charting the data, and (5) collating, summarizing, and reporting the results. 

### 2.1. Stage 1: Identifying Research Questions

The question that guided this review was formulated using the Population, Concept, and Context (PCC) framework. This framework is a structured approach commonly used in systematic and scoping reviews. It aids researchers in precisely defining the fundamental elements that shape the scope and emphasis of their study. Using the PCC framework, researchers ensure that their research question, objectives, and inclusion/exclusion criteria are meticulously defined and harmonized with the distinct aspects of the study, including the specific population, concept, and context [39].

To guide the development of this review, we formulated the following research question:

What are the current trends in rehabilitation interventions (C) for promoting balance (C) in stroke survivors (P)?

### 2.2. Stage 2: Identifying Relevant Studies

The literature search was conducted in three databases from the EBSCOhost research platform (CINAHL Complete, MEDLINE Complete, and Nursing & Allied Health Collection: Comprehensive) to identify references.

A comprehensive search strategy was developed based on the PCC framework, combining concepts in line with Medical Subject Headings (MeSH). The search algorithm applied the following combination: (Stroke) AND (rehabilitation) AND (Postural Balance).

We limited the search for “stroke” to studies where this descriptor was exclusively present in the title. This choice aimed to streamline the search process and guarantee a focused and precise selection of studies related to our research question. We intended to exclude studies involving diverse populations or topics that may have mentioned “stroke” in their abstracts or full texts without being primarily focused on it. This methodological decision was implemented to improve the relevance and specificity of the studies included in our analysis.

The PCC framework served as the foundation for developing the search strategy and establishing criteria for inclusion and exclusion (Table 1).

The search was conducted on 24 March 2023, limited to articles published in English or Portuguese and published between 2013 and 2023.

Two researchers performed the search, selection, and data extraction independently.

### 2.3. Stage 3: Study Selection

After eliminating duplicate articles, the title and abstract of the remaining search results were screened against the selection criteria. Following this, the full text of the relevant articles was reviewed, and those meeting the study criteria were included in this review.

When it was unclear whether the article fit the criteria for this review, it was automatically moved to the next phase. In cases of disagreement, a third reviewer made the final decision.

### 2.4. Stage 4: Charting the Data

Two reviewers conducted the data extraction by applying a designed instrument tailored to collect data valuable to answering the research question. The researchers collected the following data elements: general data (author’s name, publication year, title, and country), methodological details (study design and aim), and results (interventions used by health professionals to promote balance in stroke survivors). Subsequently, all authors participated in a comprehensive review and discussion of the final extraction chart.

### 2.5. Stage 5: Collating, Summarizing, and Reporting the Results

**Figure 1:**
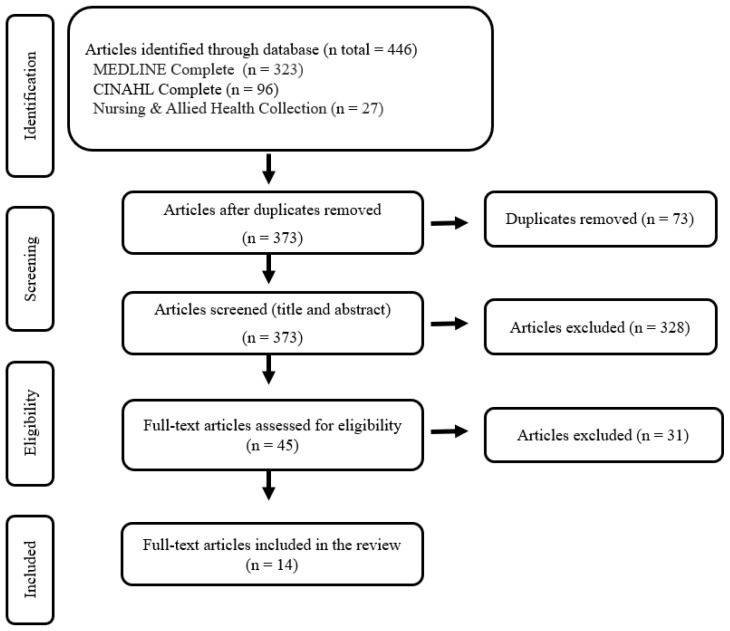
PRISMA flow chart for study selection.

The PRISMA flow diagram (Figure 1) illustrates the study identification, screening, and selection process. For data organization and summarization, a data-driven thematic analysis was utilized, following the guidelines by Braun et al. [40]. Two researchers independently conducted the data review, performing manual coding and analysis to identify recurring themes from the collected data.

## 3. Results

A total of 446 articles were identified from three databases: 323 from MEDLINE Complete, 96 from CINAHL Complete, and 27 from Nursing & Allied Health Collection: Comprehensive. After deduplicating the initial set of publications, 373 unique articles were retained. Following the screening of titles and abstracts, 328 publications were excluded. Subsequently, 45 articles underwent detailed examination, leading to the exclusion of an additional 31 articles. Consequently, 14 articles were included in the review. The flow chart describing the screening process is presented in Figure 1.

The 14 included studies were published between 2013 and 2023. Out of the 14 studies, there were 6 conducted in South Korea [31,33,34,41,42,43], 2 in Italy [44,45], 2 in India [30,46], 1 in Japan [32], 1 in Egypt [47], 1 in Thailand [48], and another in Turkey [49] (Table 2).

Data analysis revealed various interventions that promote balance in stroke survivors. The rehabilitation interventions were grouped into seven categories based on their differences and similarities. Each category is detailed below:

### 3.1. Conventional Stroke Rehabilitation Exercises

Selective trunk muscle control recovery is a strategy to improve trunk mobility, enhancing muscle coordination, postural control, and mobility in stroke survivors. Specific exercises, like “the bridge” and “the tentacle”, are effective in supine and seated positions. Complemented by conventional rehabilitation techniques, such as active mobilization, muscle strengthening, nervous system stimulation, and task-oriented activities, these exercises enhanced trunk muscle control, postural stability, balance, and mobility [45].

Another intervention used in the recovery of balance in stroke survivors was standard motor rehabilitation incorporating the affected side. This intervention comprised reflexive, synergistic, out-of-synergy movements of the paretic upper limb and lower limb; functional activities, like sit-to-stand, stepping, and reaching; and side-ward and straight-line walking. The results of this study showed that the intervention improved balance, mobility, and functionality, which were sustained three months after the intervention [46].

### 3.2. Gym-Based Interventions

A study by Wagh et al. [30] aimed to investigate the effect of core muscle strengthening using a Swiss ball on balance in stroke survivors. The intervention included conventional rehabilitation exercises, such as passive mobilization, bed mobility exercises, transfer activities, gait training, weight-bearing exercises, balance training exercises, and pelvic bridging abdominal curl-ups. Subsequently, rehabilitation with the Swiss ball was introduced, incorporating exercises in a supine position, such as pelvic bridging, unilateral bridging, and trunk rotation, with the Swiss ball. In a sitting position, participants performed static balance exercises, trunk flexion–extension, forward reach, lateral reach, and trunk flexion extension with their lower back on the Swiss ball.

Researchers concluded that core muscle strengthening with a Swiss ball and conventional stroke rehabilitation exercises was significantly more effective than relying solely on conventional stroke rehabilitation in enhancing balance among stroke survivors.

Lee et al. [31] investigated the impact of close and open kinetic chain exercises on muscle activation and balance in stroke survivors. They observed noteworthy outcomes by utilizing Leg Press Rehab and Leg Extension Rehab machines with pneumatic resistance. Close kinetic chain exercise involved seated participants extending their paretic leg on the Leg Press machine while flexing the knee from 90°. Open kinetic chain exercises were conducted facing the Leg Extension machine, with participants extending and flexing the paretic leg’s knee joint from a 90° flexed position.

In addition to conventional training, the study developed by Chang et al. [32] employed backward walking on a treadmill. The treadmill’s speed was calibrated based on participants’ prior motor function, starting at a minimum of 0.2 km/h and gradually increasing within their comfort zone. This innovative approach exhibited positive results, enhancing balance, walking speed, and cardiopulmonary fitness, augmenting the effectiveness of conventional training.

### 3.3. Vibration Therapy

Lee et al. [42] evaluated the effect of whole-body vibration therapy on balance recovery in subacute stroke survivors. The intervention was conducted using a vibratory platform (Sonic World, Wonju, Republic of Korea), where participants received vibration therapy for 30 min, in addition to conventional rehabilitation training. During the treatment, participants were seated on the vibration platform and tried to independently sit and lean on the column connected to the balance platform. Whenever the patient’s posture was about to collapse, the supervising therapist would adjust the alignment to continue the vibration therapy. Therapy occurred while the patients performed no particular activity. The study results suggest that whole-body vibration therapy showed functional improvement in the recovery of balance and daily living activities in subacute stroke survivors.

### 3.4. Rhythmic Auditory Stimulation Training

Cha et al. [41] evaluated the effectiveness of intensive gait training accompanied by rhythmic auditory stimulation. Participants received group-format rhythmic auditory stimulation training integrating personalized music tapes and a metronome; both catered to individual musical tastes. A music specialist facilitated the exercise with three songs, highlighting rhythms aligned with participants’ selected music. The metronome’s synchronization with the music’s beat heightened rhythmic perception, aligning seamlessly with participants’ walking patterns.

Participants underwent a stepwise process of rhythmic auditory stimulation training. Initially, they familiarized themselves with the music’s rhythm while coordinating their hands and feet movements to the beat. To ensure an easy transition to fitted steps, participants were instructed to walk while simultaneously synchronizing their movements with the music and the metronome.

During the training, participants engaged in intensive gait exercises with rhythmic auditory stimulation. They were guided to walk while carefully listening and matching their steps to the rhythmic cues the music and metronome provided. As the training progressed, the rhythmic auditory stimulation was gradually removed, leading participants to practice intensive gait training without external rhythmic cues. Researchers concluded that intensive gait training with rhythmic auditory stimulation improves balance and gait performance in stroke survivors.

### 3.5. Boxing Therapy

Ersoy and Iyigun [49] compared the effects of virtual and real boxing training, in addition to neurodevelopmental treatment, in stroke survivors. The neurodevelopmental approach was based on Bobath training, comprising upper extremity facilitation techniques and functional level-based activities, such as mat exercises, weight shifting training, trunk control, balance activities, and gait training. In addition, participants received either virtual boxing training through an Xbox Kinect 360 game console or real boxing training in a group format.

For virtual boxing training, the Boxing Game involved three positions: direct punch (high and low), hook (punch against the opponent’s ear), and block position (high and low). As players’ success increased (rounds completed), the opposing avatar’s reaction speed to punches intensified. In the real boxing training group, participants and the physiotherapist wore boxing mitts, following a specified treatment protocol that included unilateral and bilateral jab/direct punches (high and low), bilateral direct punches/hook punches (high and low), and bilateral punch combinations with different punching styles or sides (high and low). The findings of this present study revealed that both boxing training methods significantly improved bilateral punching time and balance functions, with no evidence of one approach being superior to the other.

### 3.6. Technology-Based Rehabilitation Interventions

Several studies introduce technology to enhance the recovery of balance in stroke survivors.

Kim et al. [34] evaluated the impact of robot-assisted trunk control training on postural control and balance. The researchers employed a specialized trunk control rehabilitation robot (3DBT-33, Man and tel, Gumi, Republic of Korea). This robot incorporated pressure sensors beneath the buttocks and feet, enabling weight distribution measurement. The training regimen included sit-to-stand exercises. For these exercises, a supporter positioned in front of the knee joint facilitated the extension of the knee joint. The chair’s seat inclination was adjustable, aiding movements involving pelvic tilt and trunk flexion. A monitor was integrated into the setup to provide feedback and motivation, offering participants a virtual reality training program.

The robot-assisted trunk control training program comprised three types of training: sitting balance, sit-to-stand, and standing balance training. Sitting balance training involved maintaining a symmetrical sitting posture by equally distributing weight on the left and right buttocks. The sit-to-stand training program was conducted sitting with the big toes positioned 10 cm behind the knee joint. A frontal supporter supported knee joint extension, while pelvic tilt and trunk flexion movements were assisted by tilting the chair’s seat angle forward. The standing balance training program mirrored the steps of the sitting balance program, but was performed in a standing position.

Researchers verified that participants who received robot-assisted training showed significant improvements in trunk control and balance.

Another study by Aphiphaksakul and Siriphorn [48] aimed to assess the impact of home-based exercise utilizing a balance disc alongside input from a smartphone inclinometer application. They employed a smartphone with the Compass Inclinometer Android OS app for their study. Securely positioned on participants’ chests via a harness and extension arm, the app displayed three inclinometers showing the smartphone’s tilt angle. During training, participants observed the app’s screen and executed movements as directed by the exercise program. Participants were seated on a chair with feet on a balance disc and performed different exercises, like leaning forward, backward, and laterally and rotating the trunk to both sides. The inclinometer was adjusted to varying degrees (15°, 30°, 45°, and 60°) to provide different challenges. The study’s results indicated that the exercise program could potentially enhance postural control and daily activity levels among stroke survivors. 

Lee et al. [33] used the Nintendo Wii Sports Resort game (Nintendo^®^, Kyoto, Japan) to explore the effects of game-based virtual reality canoe paddling training on stroke survivors. Researchers employed a chair fixed to a springboard to replicate the swaying motion experienced while canoeing. Participants performed paddling movements with both hands, holding the motion controller into a separate canoe paddle accessory. Grip-assist gloves were provided for those who had difficulty grasping the motion controller. During the intervention sessions, the study participants operated the paddle in alignment with the movements of the virtual character displayed on a TV screen. This intervention significantly improved the arm and hand’s functional performance and postural balance.

Hyun et al. [43] investigated the effects of sit-to-stand training with real-time visual feedback. Employing a motor learning approach, the training program utilized a height-adjusted leveling block without a backrest or armrest. The Wii Balance Board provided real-time visual feedback, facilitated by force plates detecting weight load, a computer, a 17-inch monitor, and a mirror. The mirror allowed participants to observe their movements instantly, aided by markers for maintaining proper alignment. The study revealed the program’s effectiveness in enhancing stroke survivors’ muscle strength, balance, gait, and overall quality of life.

In the study conducted by Morone et al. [44], researchers explored the effectiveness of balance training through a video game-based intervention in improving functional balance and reducing disability in stroke survivors. The participants engaged in balance training using the Nintendo Wii game (Nintendo^®^, Kyoto, Japan). The training involved three games enhancing balance, coordination, and endurance: hula hoop, bubble blower, and sky slalom. The study’s findings indicated that video game-based and conventional therapy improved balance and reduced disability in stroke survivors.

### 3.7. Dual-Task Aquatic and Land Motor Training

Saleh et al. [47] compared the effect of aquatic versus land motor dual-task training on the balance and gait of stroke survivors. Land motor training involves walking while holding a ball or cup and using a balance board. These tasks included various walking conditions—forward, sideways, and backward walking. The aquatic therapy group followed the same sequence of dual-task training, but performed the exercises in a swimming pool. The study’s results revealed that aquatic motor dual-task training led to significant improvements in balance and gait abilities among patients with chronic stroke compared to land motor dual-task training.

## 4. Discussion

Rehabilitation after a stroke is essential for individuals to regain their motor, sensory, and cognitive abilities. In this scoping review, we identified various rehabilitation interventions to improve balance in stroke survivors. These interventions included multiple approaches, from specific exercises focusing on trunk muscle control to using gym equipment, vibration therapy, rhythmic auditory stimulation training, boxing therapy, and technology-based rehabilitation interventions.

Conventional rehabilitation exercises are among the most accessible and practical options for stroke recovery. These interventions focus on exercises that improve trunk mobility, muscle coordination, postural control, mobility, and balance. Specific rehabilitation exercises, such as selective training of trunk muscles [45] and training of the affected side [46], have significantly improved trunk muscle coordination, postural stability, balance, and functionality in stroke survivors.

Gym-based interventions can be another accessible option and bring significant improvements to individuals. For instance, trunk muscle strengthening with a Swiss ball [30], core stability exercises [31], whole-body vibration therapy [42], and backward walking on a treadmill [32] are safe and effective options to improve physical fitness and postural balance in stroke survivors. However, they may require healthcare professionals to accompany individuals to a gym/clinic or work specifically in a space with gym equipment.

Using dual-task training in aquatic or land-based therapy has also been explored as an effective approach to improving balance and gait in stroke survivors, opening the door to combining new exercises with dual-task training. Dual-task training is an intervention with a well-documented track record of effectiveness. This approach transcends its application in stroke rehabilitation, extending its benefits to diverse populations facing neurological challenges, including individuals coping with Parkinson’s disease and dementia [50,51,52].

In the context of stroke survivors, dual-task training has emerged as a promising strategy to enhance balance and functional outcomes. This approach involves the simultaneous performance of cognitive tasks alongside motor activities, effectively mimicking real-world scenarios. This approach addresses the physical impairments associated with stroke and challenges cognitive processes, fostering improved coordination and adaptability in daily activities [47,53].

Incorporating technology into rehabilitation has opened new possibilities for improving balance and gait in stroke survivors. A set of studies explores the potential of technology in rehabilitation, including the use of a robot with a trunk control training program [11], virtual and real boxing training [49], virtual canoeing training [33], and sit-to-stand training using a mirror with real-time visual feedback [43]. These innovative interventions using virtual reality and video games in rehabilitation can be highly beneficial, with significant improvements in trunk control, balance, and muscle strength and functional improvements in upper extremities and quality of life.

Interestingly, at the foundation of each therapy described in this review, conventional rehabilitation exercises have long demonstrated their efficacy in treating stroke survivors. However, a growing trend has been developing novel forms of therapy and complementary interventions to supplement conventional training in recent years. These approaches aim to provide more diversified and innovative therapeutic options, fostering effective and engaging treatments to enhance patients’ adherence to rehabilitation plans and promote balance recovery.

Incorporating complementary interventions alongside conventional rehabilitation exercises holds promise for improving treatment outcomes for stroke survivors. These supplementary interventions can include various techniques, such as vibration therapy, rhythmic auditory stimulation training, boxing therapy, and technology-based rehabilitation interventions.

Stroke survivors face unique challenges in their recovery journey. By offering a range of therapeutic approaches, healthcare professionals can tailor treatment plans to meet the specific needs of individual patients, optimizing their chances of successful recovery [54,55,56].

Moreover, conventional rehabilitation exercises, while effective, may become monotonous and repetitive over time, leading to reduced patient engagement and motivation. Introducing novel and engaging therapies can reinvigorate patients’ interest in their rehabilitation journey, making them more likely to actively participate in their treatment and adhere to the prescribed exercise regimens [57]. Enhanced patient engagement can significantly impact the overall success of rehabilitation efforts, as consistent and committed participation can lead to better and faster recovery outcomes [58,59].

Additionally, innovative therapeutic options can tap into different aspects of the patient’s physical and cognitive abilities, stimulating various neural pathways and promoting neural plasticity. By challenging the brain and body in new ways, these therapies may help rewire neural connections and facilitate relearning lost motor skills and balance control [60,61,62]. Furthermore, a diversified range of therapeutic options can also cater to the preferences and interests of patients, making the rehabilitation process more enjoyable and personalized. When patients find their therapy sessions enjoyable and rewarding, they are more likely to stay committed to their treatment plan, leading to better compliance and improved outcomes [58,59,63,64].

Overall, this review offers valuable information on the various interventions used by healthcare professionals to improve the balance of stroke survivors. Stroke is a complex medical condition that often leads to impaired balance, which can have a significant impact on a person’s quality of life and functional independence [6,7]. Therefore, understanding and addressing balance deficits in stroke survivors is crucial in their rehabilitation journey.

One of the standout contributions of this review lies in its ability to identify various interventions that can be employed to rehabilitate balance in stroke survivors.

The systematic categorization of these interventions is particularly advantageous, as it provides healthcare professionals with a clear roadmap. These professionals often face the challenge of selecting the most appropriate intervention strategies tailored to their patient’s needs and conditions. This categorization simplifies selecting the most suitable interventions for their clients, enabling personalized and evidence-based practice.

This review underscores the potential of technology-assisted interventions in an era where technology is increasingly integrated into healthcare. It sheds light on the benefits of incorporating virtual reality, smartphone applications, gaming systems, and other technological tools into balance rehabilitation. This keeps healthcare practices up-to-date and offers innovative avenues for improving post-stroke balance recovery.

### Strengths and Limitations

One of this review’s primary strengths is its targeted focus on experimental studies, which enables a clear and focused investigation of current trends in balance rehabilitation for stroke survivors. This review provides valuable insights into promising approaches for balance rehabilitation in stroke survivors by explicitly identifying and analyzing interventions rigorously tested through experimental studies. The comprehensive overview of these interventions allows for a thorough understanding of the specific techniques and methodologies employed, facilitating their replication and implementation by the rehabilitation workforce.

However, this research does have some limitations. Focusing solely on experimental studies may result in overlooking valuable insights from non-experimental studies that could contribute to a more comprehensive understanding of balance rehabilitation interventions. Moreover, restricting the search to the EBSCOhost research platform might have excluded pertinent studies from other sources. Additionally, the decision to narrow the search by including only studies with the term “stroke” in the title could potentially limit the breadth of relevant research, as studies that mentioned “stroke” in their abstracts or full texts without it being in the title might have been missed. Furthermore, specific decision criteria, like excluding non-English and non-Portuguese language papers, may introduce biases by excluding relevant literature. It is essential to acknowledge these limitations to provide a balanced interpretation of the findings and to encourage further research exploration beyond the scope of this review.

## 5. Conclusions

This scoping review highlights the diversity and potential of various rehabilitation interventions to address stroke survivors’ balance challenges. From conventional rehabilitation exercises to gym-based interventions, vibration therapy, rhythmic auditory stimulation training, boxing therapy, dual-task training, and technology-based rehabilitation interventions, each approach offers unique benefits and has the potential to significantly contribute to the overall well-being and functional recovery of stroke patients.

Considering these findings, healthcare professionals are encouraged to design rehabilitation programs for stroke survivors based on their specific needs and preferences. This review’s systematic categorization of interventions serves as a practical framework for customizing rehabilitation plans.

Moreover, it is worth emphasizing dual-task training as a means to effectively address both motor and cognitive aspects of rehabilitation and enhance motivation and adherence levels among stroke survivors.

The integration of technology-assisted interventions shows great promise in balance rehabilitation. Innovative tools, like virtual reality, smartphone applications, and gaming systems, have not only proven their efficacy, but also have the potential to enhance motivation and engagement among stroke survivors participating in rehabilitation programs.

Future research should study the long-term effects of these interventions, exploring their sustainability and enduring benefits over time. Additionally, conducting comparative studies between different intervention modalities can yield valuable insights into their relative effectiveness, further advancing the field of stroke rehabilitation.

## Figures and Tables

**Table 1:** Inclusion/exclusion criteria.

Parameter	Inclusion Criteria	Exclusion Criteria
Population	Stroke survivors; Adults ≥ 18 years old.	Other health conditions besides stroke; Participants < 18 years old.
Concept	Studies that explore non-invasive interventions that promote balance.	Studies that do not address interventions that promote balance; Studies that use invasive techniques.
Context	Studies conducted in rehabilitation settings (e.g., acute, post-acute, and long-term care).	Studies conducted in non-healthcare or non-rehabilitation settings.
Study design	Experimental studies and quasi-experimental studies.	Other type of studies.

**Table 2:** Data extraction and synthesis.

Author/Year/Title/Country	Study Design/Study Aim	Intervention
Lee et al. [31] (2013) The effects of closed and open kinetic chain exercises on lower limb muscle activity and balance in stroke survivors South Korea	Randomized controlled trial To examine the effects of close kinetic chain exercise and open kinetic chain exercise on muscle activation of the paretic lower limb and balance in chronic stroke subjects.	Gym-based interventions
Cha et al. [41] (2014) Intensive gait training with rhythmic auditory stimulation in individuals with chronic hemiparetic stroke: a pilot randomized controlled study South Korea	Randomized controlled trial To investigate the effect of intensive gait training with rhythmic auditory stimulation on postural control and gait performance in individuals with chronic hemiparetic stroke.	Rhythmic auditory stimulation training
Morone et al. [44] (2014) The efficacy of balance training with video game-based therapy in subacute stroke patients: a randomized controlled trial Italy	Randomized controlled trial To investigate the efficacy of balance training using video game-based intervention on functional balance and disability in individuals with hemiparesis due to stroke in subacute phase.	Technology-based rehabilitation interventions
Pandian et al. [46] (2014) Does motor training of the nonparetic side influences balance and function in chronic stroke? A pilot RCT India	Randomized controlled trial To examine the effect of a motor therapy program primarily involving the nonparetic side on balance and function in chronic stroke.	Conventional stroke rehabilitation exercises
Lee et al. [42] (2017) The effect of a whole-body vibration therapy on the sitting balance of subacute stroke patients: a randomized controlled trial South Korea	Randomized controlled trial To evaluate the effects of a whole-body vibration therapy on recovery of balance in subacute stroke patients who were unable to gain sitting balance.	Vibration therapy
Wagh et al. [30] (2017) Effectiveness of Core Muscle Strengthening with Swiss Ball on Balance in Cerebellar Stroke India	Experimental study To find out effect of core muscle strengthening with Swiss ball on balance in cerebellar stroke patients.	Gym-based interventions
Lee et al. [33] (2018) Game-Based Virtual Reality Canoe Paddling Training to Improve Postural Balance and Upper Extremity Function: A Preliminary Randomized Controlled Study of 30 Patients with Subacute Stroke South Korea	Randomized controlled trial To investigate the effects of game-based virtual reality canoe paddling training, when combined with conventional physical rehabilitation programs, on postural balance and upper extremity function in 30 patients with subacute stroke.	Technology-based rehabilitation interventions
Saleh et al. [47] (2019) Effect of aquatic versus land motor dual task training on balance and gait of patients with chronic stroke: A randomized controlled trial Egypt	Randomized controlled trial To compare the effect of aquatic versus land motor dual-task training on balance and gait of patients with chronic stroke.	Dual-task aquatic and land motor training
Bigoni et al. [45] (2021) Retraining selective trunk muscle activity: A key to more successful rehabilitation outcomes for hemiparetic stroke patients Italy	Quasi-experimental study To investigate if the inclusion of specific exercises for the trunk muscles in a rehabilitation program for chronic hemiparetic patients could lead to an additional improvement.	Conventional stroke rehabilitation exercises
Chang et al. [32] (2021) The Effect of Walking Backward on a Treadmill on Balance, Speed of Walking and Cardiopulmonary Fitness for Patients with Chronic Stroke: A Pilot Study Japan	Randomized controlled trial To determines the effect of walking backward on a treadmill on balance, speed of walking, and cardiopulmonary fitness for patients with chronic stroke.	Gym-based interventions
Ersoy & Iyigun [49] (2021) Boxing training in patients with stroke causes improvement of upper extremity, balance, and cognitive functions but should it be applied as virtual or real? Turkey	Randomized controlled trial To compare the effects of virtual and real boxing training, in addition to neurodevelopmental treatment, on the upper extremity, balance, and cognitive functions in hemiparetic stroke patients.	Boxing therapy
Hyun et al. [43] (2021) The Effects of Sit-to-Stand Training Combined with Real-Time Visual Feedback on Strength, Balance, Gait Ability, and Quality of Life in Patients with Stroke: A Randomized Controlled Trial South Korea	Randomized controlled trial To investigate the effects of lower limbs muscles’ strength, balance, walking, and quality of life through sit-to-stand training combined with real-time visual feedback in patients with stroke and to compare the effects of classic sit-to-stand training.	Technology-based rehabilitation interventions
Aphiphaksakul & Siriphorn [48] (2022) Home-based exercise using balance disc and smartphone inclinometer application improves balance and activity of daily living in individuals with stroke: A randomized controlled trial Thailand	Randomized controlled trial To determine the effects of home-based exercise utilizing a balance disc with input from a smartphone inclinometer application on sitting balance and activities of daily living in stroke survivors.	Technology-based rehabilitation interventions
Kim et al. [34] (2022) Effects of robot-assisted trunk control training on trunk control ability and balance in patients with stroke: A randomized controlled trial South Korea	Randomized controlled trial To evaluate the effects of robot-assisted trunk control training on trunk postural control and balance ability in stroke patients.	Technology-based rehabilitation interventions

## Data Availability

The data presented in this study are available on request from the first author.
